# Risk Factors of Post-Traumatic Stress Disorder (PTSD) after Wenchuan Earthquake: A Case Control Study

**DOI:** 10.1371/journal.pone.0096644

**Published:** 2014-05-06

**Authors:** Yongzhong Cheng, Fang Wang, Jin Wen, Yingkang Shi

**Affiliations:** 1 Department of Hospital Management and Health Policy, West China Hospital, Sichuan University, Chengdu, P.R. China; 2 Department of Operation Management, West China Hospital, Sichuan University, Chengdu, P.R. China; 3 Shuangliu Center for Disease Control and Prevention (CDC), Shuangliu, Chengdu, P.R. China; Univ of Toledo, United States of America

## Abstract

**Background:**

Few clues were found in the literature about the independent risk factors for PTSD among earthquake survivors in Sichuan province three years after the 2008 earthquake. Ours was the first case-control study with matching factors of age and distance from the epicenter among survivors age 16 years or older, three years after the catastrophe.

**Objectives:**

To identify independent risk factors for PTSD among earthquake survivors.

**Methods:**

We performed a population-based matched case-control study. The cases were drawn from earthquake areas three years after the Wenchuan earthquake, including 113 cases who met positive criteria for PTSD symptoms according to the PCL-C (PTSD Checklist-Civilian Version) score and 452 controls who did not meet the criteria. Cases and controls were matched individually by birth year (+ three years) and the town they lived in when the earthquake occurred.

**Results:**

Independent risk factors for PTSD symptoms included two-week disease prevalence (odds ratio [OR],1.92; 95% confidence interval [CI],1.18–3.13), witnessing someone being killed in the earthquake (OR, 2.04;95%CI, 1.17–3.58), having no regular income after the earthquake (OR, 0.52; 95%CI, 0.28–0.98), receiving mental health support only one time after the earthquake (OR, 2.43; 95%CI, 1.09–5.42) and lower social support (lower PSSS score) (OR, 0.95; 95%CI, 0.93–0.97).

**Conclusion:**

Earthquake experience, suffering from physical illnesses, lack of stable income, and lower social support were associated with PTSD symptoms.

## Introduction

The Ms 8.0 Wenchuan earthquake of May 12, 2008—the strongest earthquake since the establishment of People's Republic of China (PRC)—has caused great life and financial losses. Tens of thousands of people lost their homes and families. According to the Ministry of Civil Affairs, as of August 25, 2008, there were 69 226 people killed, 374 643 injured, and 17 923 missing. Immediately after the disaster, the Chinese government took several positive measures, such as providing financial support from the central government and 19 local provinces for reconstruction of the destroyed areas. Post-disaster reconstruction, planned to be complete within three years, was actually finished ahead of schedule in May 2011, according to claims by Wen Jiabao, the Prime Minister of China [1].

People who have survived a catastrophe such as an earthquake may develop psychological trauma and psychiatric disease, including post-traumatic stress disorder (PTSD). PTSD is a common mental health problem among earthquake survivors in China[2], and it may last for a long time. For example, Zhang reported that the prevalence rate of probable PTSD was 26.3% one year after the Wenchuan earthquake [3]. Zhao baoJia[4]estimated the prevalence of PTSD to be 12.4% among child survivors ages 8 to 16 years, 15 months after the earthquake. Jin Wen [5] documented that prevalence of PTSD symptoms was 8.8% among hard-hit area survivors 3 years after the Wenchuan earthquake.

With the completion of post-Wenchuan earthquake reconstruction in 2011, the survivors' houses are equal to or even better than their house before the disaster, but current evidence reveals that there is still a high prevalence of PTSD among survivors. To provide appropriate interventions for reducing the occurrence of PTSD, we must identify the risk factors for the mental impairment. Several previous articles showed some possible contributing factors for disaster related PTSD which could be divided into five categories: (1) demographic variables, such as female, younger age, lower socioeconomic status, minority, educational and intellectual disadvantage [3], [6], [7], [8], [9]; (2) pre-disaster factors, such as psychiatric history, previous adversity, negative parenting experiences, and early traumatization. [6], [10]; (3) disaster related factors, such as trauma severity, family member death or missing, property damage, and witnessing someone being killed in the disaster [6], [8], [7], [2], [11]; (4) post-disaster factors, such as lack of social support, living in a shelter or temporary house, lower household income and other life stress [6], [7], [8], [9], [11], [12]; and (5) other factors, such as elevated norepinephrine and, chronically low levels of cortisol [13], [14].

Unfortunately, little evidence coming from local communities revealed the contributors of chronic PTSD, and most of the previous PTSD-related studies were cross-sectional, focusing on special populations such as students, injured, soldiers, and doctors and nurses who were involved in earthquake relief efforts. Therefore, we designed this case control study to identify independent risk factors for PTSD. To reveal the personal factors and factors directly related to the earthquake experience and relief, this study focused on demographic, earthquake related and post-earthquake variables.

## Methods

### Ethics statement

The Institutional Review Board (IRB) of West China Hospital in Sichuan University approved this study. Due to the illiteracy of many of the people in the earthquake-affected areas, written consent is not common practice and may violate confidentiality. Therefore, a consent form to obtain verbal consent from respondents was proposed and approved by the IRB, together with the study protocol. Prior to the interview, each investigator read carefully to the interviewee the consent form, which contains information on the objectives of the study, the selection process, risks, benefits and freedom of the participation, as well as information on confidentiality [5].

### Study design and setting

This was a matched case-control study, and the sample was selected from 22 townships of 6 counties in Sichuan province from May to July 2011, three years after the Wenchuan earthquake. More detailed sampling information is available in Jin's paper [5]. After investigating 2525 earthquake survivors, cases and controls were determined according to scores from the PTSD Checklist-Civilian Version (PCL-C). Hence, case and control status was blind for investigators.

### Cases and controls

The PTSD Checklist-Civilian Version (PCL-C) was used to screen for symptoms of earthquake related PTSD. PCL-C is a 17-item(all the items were predefined as the earthquake related ones when performing the survey) self-report symptom scale that corresponds to DSM-IV criteria[15], and its total score ranges from 17 to 85. Evidence shows that the scale has good validity, reliability and accuracy for PTSD screening[16]. The Internal consistency (Cronbach's Alpha coefficient) of the PCL-C in our study was 0.80. Subjects with a score of 38 or higher were classified as having probable PTSD [17], [18]and then were labeled as cases. Controls were selected from earthquake-related survivors whose PCL-C scores were less than 38, in a ratio of 4∶1 to cases, and were matched for township and age (± 3 years of age).

### Variables and measurements

General demographic information(age, gender, nationality, education, household income, house location before earthquake) and other factors (mahjong playing, two-week disease prevalence rate, chronic disease rate, experience during earthquake(buried or injured, witnessing others being injured or killed),having relatives injured or killed during the earthquake, displacement after the earthquake, and the number of mental health support sessions received after the earthquake) were collected using a predefined questionnaire.

Chronic disease was defined as at least one of the following illnesses [5]: chronic non-specific lung disease (asthma, bronchitis and pulmonary emphysema), cardiac diseases, atherosclerotic disease, cerebrovascular disease (stroke, excluding transient ischemic attacks), diabetes mellitus, malignant neoplasm, osteoarthritis, and rheumatoid arthritis. All the above chronic diseases should have been diagnosed by physicians in hospitals or clinics before the investigation and be with current symptoms or treatments. Two-week disease prevalence was investigated by asking the interviewees whether members of their household had been ill in the previous two weeks, and was measured in terms of the number of those ill in the past two weeks. Family members injured or killed during the earthquake were categorized one of two ways, according to their relationship with subjects: first-class relatives(closer family members including mother/father, mate, and son/daughter)and second-class relatives (less close family members including brothers/sisters, grandfather/grandmother, grandson/granddaughter and other relatives).

Perceived social support was measured using Perceived Social Support Scale(PSSS) [19], a 12-item self-report scale with a total score ranging from 12 to 60, which assesses perceived availability of and satisfaction with support received from family, friends and other special person with whom one can share his or her joys and sorrows. Note that a higher score means a higher degree of perceived social support. The Internal consistency (Cronbach's Alpha coefficient) for this sample was 0.85.

### Bias

Our investigators consisted of professors and post-graduate students from West China Hospital and West China School of Public Health, Sichuan University. They were well-trained on the questionnaire and investigation techniques, including mutual communication with participants by dialect.

### Statistical methods

Frequencies, percentages, means and standard deviations were calculated for descriptive analysis. Conditional logistic regression analysis was used to compare cases with their matched controls for PTSD risk factors in both univariate and multivariate analysis. Odds ratio (OR) of the variables were assessed and 95% confidence intervals (CI) of ORs were calculated. The level of statistical significance was less than 0.05 in this study. Data were analyzed with SPSS version 17·0 (SPSS Inc, Chicago, Illinois) and Stata version 10.0 (StataCorp, College Station, TX, USA).

## Results

**Figure 1:**
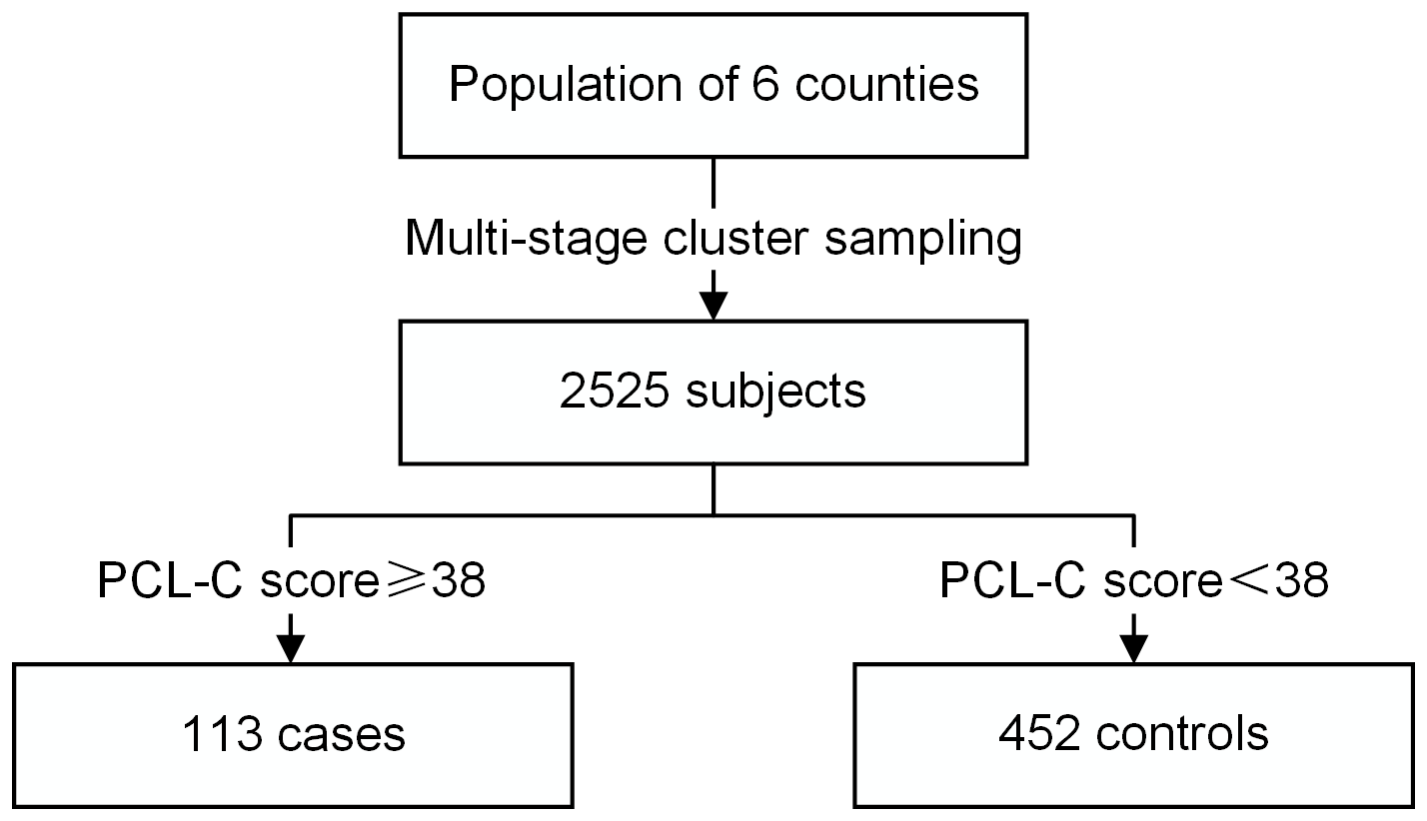
Flow chart of sampling.

Of the 2525 eligible respondents, 113 cases were identified and 452 controls were individually matched to the cases (Figure 1). All of the 565 subjects completed the questionnaire with no missing data.32.74% of cases and 43.81% of controls were male. The two-week disease prevalence and chronic disease prevalence of cases were 40.71% and 45.13% respectively, both higher than those of controls. There were more people in the case group than the control group who witnessed someone being injured or killed during earthquake. A higher number of cases than controls had first-class relatives who died or went missing during the earthquake. The percentage of people who had a regular income after the earthquake was lower for cases than for controls. Of the cases, 12.39%received mental health support one time, and 21.24% received mental health support twice after earthquake, compared to 5.79% and 18.36% respectively for the controls (Table 1).The mean score(standard deviation (SD)) of PSSS was 55.69(13.46)for cases and 62.33(11.11)for controls, respectively.

**Table 1:** Factors related to cases and controls.

	Cases(n = 113)		Controls(n = 452)	
	No.	%	No.	%
**Gender**				
Male	37	32.74	198	43.81
Female	76	67.26	254	56.19
**Nation**				
Han ethnic	106	93.81	422	93.36
Minority	7	6.19	30	6.64
**Education**				
Higher than primary school	39	34.51	147	32.52
Primary school or lower	74	65.49	305	67.48
**Playing Mahjong**				
Yes	32	28.32	126	27.88
No	81	71.68	326	72.12
**Two-week disease**				
Yes	46	40.71	123	27.21
No	67	59.29	329	72.79
**Chronic disease**				
Yes	51	45.13	196	43.36
No	62	54.87	256	56.64
**Buried or injured in earthquake**				
Yes	20	17.70	51	11.28
No	93	82.30	401	88.72
**Witnessing someone being injured in earthquake**				
Yes	83	73.45	325	71.90
No	30	26.55	127	28.10
**Witnessing someone being killed in earthquake**				
Yes	74	65.49	258	57.08
No	39	34.51	194	42.92
**Family member injured during earthquake**				
Yes	49	43.36	169	37.39
No	64	56.64	283	62.61
**Family member died or went missing during earthquake**				
First class relatives	33	29.20	87	19.25
Second class relatives	25	22.12	113	25.00
No	55	48.67	252	55.75
**Family income after earthquake(RMB)**				
<5000	20	17.70	91	20.13
5000∼20000	70	61.95	230	50.88
>20000	23	20.35	131	28.98
**Having regular income after earthquake**				
Yes	23	20.35	148	32.74
No	90	79.65	304	67.26
**Displacement after earthquake**				
Yes	36	31.86	104	23.01
No	77	68.14	348	76.99
**Times of mental health support received after earthquake**				
None	75	66.37	342	75.66
1 time	14	12.39	27	5.79
2 or more times	24	21.24	83	18.36

Sixteen factors were tested by univariate analysis as potential risk factors for PTSD. Being female, two-week disease prevalence, having first class relatives who died or went missing during the earthquake, having no regular income after the earthquake, displacement after the earthquake, receiving mental health support only one time after the earthquake, and lower PSSS score were all significantly associated with higher PTSD symptoms prevalence. (Table 2)

**Table 2:** Results of conditional logistic regression.

	Univariate estimate			Multivariate estimate		
	OR	95%CI	P	OR	95%CI	P
**Gender**						
Male						
Female	1.64	1.05–2.56	0.031	1.51	0.89–2.55	0.128
**Nation**						
Han ethnic						
Minority	1.10	0.43–2.83	0.849			
**Education**						
Higher than primary school						
Primary school or lower	0.88	0.52–1.48	0.632			
**Playing Mahjong**						
No						
Yes	1.024	0.63–1.66	0.921			
**Two-week disease**						
No						
Yes	1.86	1.20–2.87	0.005	1.92	1.18–3.13	0.009
**Chronic disease**						
No						
Yes	1.09	0.69–1.71	0.713			
**Buried or injured in earthquake**						
No						
Yes	1.73	0.97–3.08	0.064	1.38	0.72–2.65	0.326
**Witnessing someone being injured in earthquake**						
No						
Yes	1.11	0.65–1.89	0.708			
**Witnessing someone being killed in earthquake**						
No						
Yes	1.55	0.96–2.50	0.074	2.04	1.17–3.58	0.012
**Family member injured during earthquake**						
No						
Yes	1.31	0.85–2.02	0.226			
**Family member died or went missing during earthquake**						
No						
Second class relatives	1.08	0.62–1.89	0.790	1.04	0.56–1.94	0.898
First class relatives	1.95	1.12–3.39	0.018	1.64	0.88–3.04	0.117
**Family income after earthquake (RMB)**						
<5000						
5000∼20000	1.41	0.80–2.48	0.240			
>20000	0.79	0.40–1.56	0.498			
**Having regular income after earthquake**						
No						
Yes	0.49	0.29–0.82	0.007	0.52	0.28–0.98	0.041
**Displacement after earthquake**						
No						
Yes	1.72	1.04–2.84	0.035	1.52	0.79–2.50	0.247
**Times of mental health support received after earthquake**						
None						
1 time	2.52	1.24–5.15	0.011	2.43	1.09–5.42	0.029
2 or more times	1.42	0.81–2.50	0.214	1.34	0.73–2.50	0.352
**PSSS Score**	0.95	0.94–0.97	<0.001	0.95	0.93–0.97	<0.001

Multivariable conditional logistic regression analysis was then performed. Two-week disease prevalence(odds ratio(OR = 1.92; 95%CI = 1.18to 3.13), witnessing someone being killed in the earthquake(OR = 2.04; 95%CI = 1.17 to 3.58), having no regular income after the earthquake(OR = 0.52; 95%CI =  0.28 to 0.98), receiving mental health support only one time after the earthquake(OR = 2.43; 95%CI = 1.09 to 5.42), and lower PSSS score (OR = 0.95; 95%CI = 0.93 to 0.97) were revealed to be independent risk factors for PTSD symptoms (Table 2).

## Discussion

This population-based case-control study, including 113 cases and 452 controls, demonstrated that a number of risk factors were responsible for PTSD. Two-week disease prevalence, witnessing someone being killed in the earthquake, having no regular income after the earthquake, receiving mental health support only one time after the earthquake, and lower social support were all identified to be independent risk factors for PTSD symptoms.

Interestingly, receiving mental health only one time was associated with a 2.43-fold increase in risk for PTSD, while receiving no mental health support or two or more sessions of mental health support were without significant association with PTSD in either univariate analysis or multivariate analysis. This finding differs from other literatures that claim that victims with psychological help are less prone to express PTSD[4], [20]. A probable reason for this difference is that after the Wenchuan earthquake, many volunteers went to disaster areas to provide psychological assistance. Many of the volunteers were college students, social workers and other people without professional knowledge of psychology or specific psychological counseling skills. Furthermore, there was a series of aftershocks and lack of living resources in the following few months, making it difficult for survivors and supporters to engage in long-term counseling. Hence, survivors who received mental health support only one time simply recalled miserable memories and re-experienced terrible scenes without sufficient help to release and overcome the negative emotions, which may have led to secondary psychological trauma. Providing long-lasting and available mental health support after a disaster is a meaningful contribution to PTSD prevention and recovery. Our study was the first to identify one-time mental health support after the earthquake as an independent risk factor for PTSD. This extremely interesting finding needs to be confirmed by future disaster research.

Two-week disease prevalence was significantly associated with PTSD symptoms, which indicates people with poor physical health might be at risk for developing PTSD. Similar findings were revealed by another study [21].The existing evidence shows that PTSD is associated with poor self-reported health and increased utilization of medical services[22]. Likewise, PTSD symptoms may predispose affected persons to increased risk for medical problems [23]. Thus, after an earthquake, governments should supply both medical service and mental health support to earthquake survivors to lower the prevalence of PTSD symptoms.

Some studies reported that lower income is associated with poor mental health [8]. Our study demonstrated that household income had no significant association with PTSD, while having no regular income after the earthquake increased the risk of PTSD. Most of the survivors, especially the elderly, were farmers who had farmed on their land for their entire life, but then unfortunately lost their houses and land during the earthquake. Due to the fact that most hard-hit regions were remote areas with mountains, it was impossible for local survivors to obtain new land. Although they re-built their homes and townships with the help of the government and social organizations shortly after the earthquake, some of them found that it was hard to find a regular job to support their families. These factors, along with fear about the future to some extent, made some survivors suffer more negative emotions, which may have increased the risk of developing PTSD.

Perceived social support refers to the respondents' subjective sense of social support. A lower PSSS score means subjects felt less social support from family, friend and special person with whom one can share his or her joys and sorrows. Our study determined lower social support was a significant predictive factor for PTSD symptoms, which is consistent with other studies [9], [11], [12], [24].Witnessing someone being killed in earthquake independently predicted PTSD, which was also documented in previous reports[2], [11], [20], [25].

Some studies[3], [8], [9], [11], [26], [27] pointed out that females are prone to showing more PTSD symptoms, while other studies [20]reported that male students were more prone than female students to develop PTSD. In our study, univariate analysis showed that females had a significantly higher risk for PTSD symptoms, but being female proved to not be an independent risk factor for PTSD. Previous literature reported that experiencing the death of a close family member was the major risk factor for PTSD in children [4], [28] and adults[2], [8].However, in this study, having a close family member die or go missing contributed to PTSD prevalence in adults, but was not an independent risk factor for PTSD either. Unlike for adults, for children, “close family members” mostly refers to their parents, whose death means quite a lot for them. Moreover, children do not possess the mental maturity that adults do to deal with such psychological trauma. This may lead to the difference in contribution of this factor to PTSD between children and adults. There may exist multicollinearity between witnessing death in the earthquake and having close family die, and the two literatures [2], [8] mentioned above did not consider the factor of witnessing a death.

Some recent published articles [25], [29], [30] also explored the determinants of PTSD among Wenchuan earthquake survivors. Kun P. et al [29] reported that older age, female gender, unmarried/divorced/widowed, ethnic minority, death of family member, no household income and damaged household were independent risk factors for PTSD symptoms in heavily damaged areas. Guo J. et al [30] demonstrated that female gender, being married, low education, non-drinking, and poor self-perceived health status were significantly associated with PTSD during the early period following the earthquake; and depression was significantly associated with survivors' PTSD throughout the 44 months period after the earthquake. Zhou X. et al [25] revealed that risk factors for PTSD included old age, female gender, living alone, buried in the earthquake, injured in the earthquake, operated on after the earthquake, witnessing someone get injured in the earthquake, witnessing someone get buried in the earthquake, and witnessing someone die in the earthquake. While some factors reported above are similar to the results from our study, this study showed some other important determinants (eg. lower social support) for PTSD symptoms. However, simply comparing our findings with those of the above articles is not rational for the following concerns. On one hand, Kun P. and Zhou X.’ s studies were conducted within 3–6 months, a very short period after the earthquake, while ours was performed 3 years post the disaster. The long term impact of a catastrophe on health is often ignored and different from short term effect. Moreover, we do need solid evidence for better management for the survivors over a long period of time. On the other hand, all the above three papers were cross-sectional study whilst this study was a case control design. It is well known that case control study is better than cross sectional study when exploring a causal relationship. Nevertheless, varying studies with different findings at different time point jointly contributed to the development of disaster epidemiology and the complete understandings of PTSD development after an earthquake.

Certain limitations of the study should be recognized. First of all, we failed to analyze the impact of age and distance from the epicenter of all subjects' home because of matching. Second, although widely used in the world, PCL-C is a screening measurement of PTSD, not a diagnostic tool. PCL-C was used because in such a large population-based survey, it costs too much time and money to use a formal diagnostic tool. Hence, some of the control and cases subjects may be incorrectly grouped, which would lead to a bias in our results. Third, although we collected data on the number of times of mental health support received, many subjects could only give imprecise information because much time had passed since the earthquake. Therefore, we can only divide the times of mental health assistance into three categories instead of treating this variable as a continuous variable.

## Conclusions

In summary, this paper revealed risk factors for PTSD among adult earthquake survivors and estimated the magnitude of risk associated with each factor. Our study discussed the relationship between receiving mental health support and PTSD prevalence and provided further support for other risk factors. For many of these risk factors, having a regular income after the earthquake is a clear factor for preventing PTSD. There should be more consideration about providing more job opportunities for earthquake survivors in reconstruction efforts.
